# Female Athlete Triad and Relative Energy Deficiency in Sport (REDs): Nutritional Management

**DOI:** 10.3390/nu16030359

**Published:** 2024-01-25

**Authors:** Monika Grabia, Jakub Perkowski, Katarzyna Socha, Renata Markiewicz-Żukowska

**Affiliations:** Department of Bromatology, Faculty of Pharmacy with the Division of Laboratory Medicine, Medical University of Białystok, 2D Mickiewicza Street, 15-222 Białystok, Polandkatarzyna.socha@umb.edu.pl (K.S.);

**Keywords:** female athlete triad, relative energy deficiency, energy availability, bone mineral density, menstrual disorders, nutrition, nutrient intake, bioavailability

## Abstract

The female athlete triad (TRIAD) is a spectrum of disorders involving low energy availability (LEA), low bone mineral density, and menstrual disorders. It is increasingly common to use the term ‘relative energy deficiency in sport’ (RED), emphasising the extensive impact of LEA on the body. The aim of this narrative review was to gather original research encompassing female athletes across various sports as well as to collect findings on the potential of a nutrition-focused approach to prevent or treat the aforementioned disorders. A comprehensive search was conducted in PubMed and Scopus. Several challenges were identified regarding the adequacy of the energy availability, protein, and carbohydrate requirements in the diets of female athletes. Moreover, insufficient intake of vitamin D has been observed across all athlete groups studied. This insufficiency also extends to the average requirement for Ca, Mg, the Ca/P ratio, Zn, and Fe. To address those concerns, a nutritional approach is proposed in the latter part of this review. The factors that can improve the absorption of micronutrients have also been discussed. The TRIAD/REDs affect an ever-growing number of women and require appropriate therapeutic management, particularly through nutritional care. Therefore, cooperation within an interdisciplinary team comprising a physician, nutritionist, physiotherapist, and psychologist is crucial.

## 1. Introduction

The female athlete triad (TRIAD) was formerly defined as a cluster of symptoms related to low energy availability (LEA), either with or without coexisting nutritional disorders (including anorexia nervosa), menstrual dysfunction (particularly secondary amenorrhea), and low bone mineral density (BMD) [1]. Currently, the International Olympic Committee, through a consensus, has introduced the term ‘relative energy deficiency in sport’ (REDs) as the new term to emphasise the fact that a variety of health issues (impairments in the physiological and/or psychological state of the body) resulting from LEA may affect not only female but also male athletes [2]. The presence of significant anatomical and physiological differences between the genders, notably hormonal intricacies, means that the risks of developing health disorders vary between men and women [3,4]. Therefore, for the purpose of this review, the focus will be on women and the most common health concerns caused by LEA. The disciplines that have been studied to identify at-risk groups primarily involve women who practise gymnastics, running, skating, and ballet. However, it is also increasingly common to observe those issues among women participating in leisure sports [1].

Appropriate nutrition for athletes not only enhances sports performance but also safeguards them against injuries and health deterioration [5,6]. Sports nutrition presents a major challenge for sports mentors and athletes themselves. Hence, it is imperative to emphasise the critical role of proper nutritional programming, not only for an athlete’s preparation before events, but also for preventive measures (to mitigate the incidence of adverse health outcomes) and therapeutic action (addressing the nutritional management of LEA’s effects). Supporting athletes to retain or regain their health and athletic potential is crucial for the future years of their sports careers [5,7]. The scientific literature contains papers that describe the problem of the TRIAD/REDs and therapeutic approaches based on medical treatment. However, there is a noticeable absence of publications that comprehensively compile and present the pivotal importance of a proper nutritional approach in the prevention or treatment of those disorders. 

Consequently, the aim of the study has been to gather and summarise relevant literature in a narrative review focusing on nutritional management concerning the emergence of the TRIAD/REDs. This review discusses the research conducted on women engaged in various sports, evaluating parameters, such as exercise energy expenditure (EEE) and energy availability (EA) as well as the intake of macronutrients and micronutrients crucial for maintaining proper bone density (vitamin D, calcium, phosphorus, magnesium, zinc, and iron). Furthermore, a proposed nutritional approach has been outlined to address each component of the TRIAD. The review also provides a detailed description of those essential nutrients, emphasising their relevant roles in sports and the disorders in question. Furthermore, it discusses their dietary sources and provides insights into factors that may either impair or enhance their absorption.

## 2. Materials and Methods

**Figure 1:**
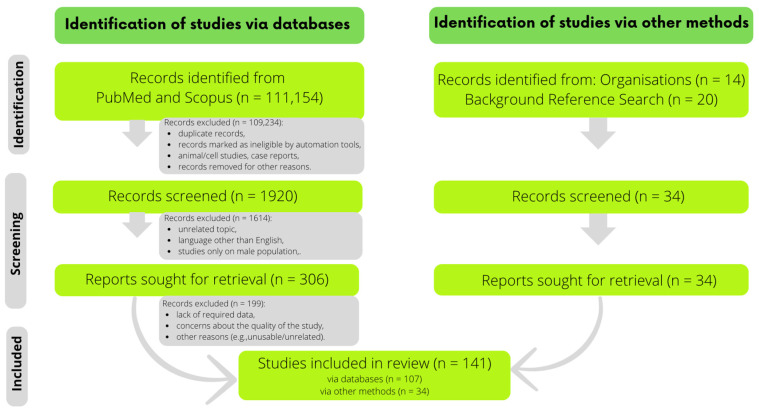
The selection process on the flowchart.

The search for publications was conducted across PubMed and Scopus. The primary keywords used included: ‘female athlete triad’, ‘relative energy deficiency in sport’, ‘female athlete + chosen micronutrient’, ‘physical activity or female athletes + chosen micronutrient’, ‘chosen micronutrient + supplementation + sport’, ‘chosen micronutrient + bioavailability’, ‘chosen micronutrient + absorption’, ‘female athlete microelements’, ‘female athlete serum levels’, and ‘sports nutrition’. Initially, over 110,000 records were identified, out of which 340 publications were reviewed and pre-selected and retrieved, and 142 were ultimately included in the review. The focus was on original or review papers, mainly regarding women practicing various sports. The exclusion criteria were language other than English, animal/cell studies, and case reports. The primary objectives of the narrative review were to discuss EA and EEE levels, examine macronutrient and micronutrient intake, present the results of various interventions aimed at mitigating health effects associated with REDs, and propose nutritional management strategies for REDs. For this purpose, data such as the type of sport discipline, EA, EEE, and macronutrient and micronutrient intake were extracted from relevant studies. If studies reported those parameters in different units, recalculations were performed wherever it was possible. Interventions were assessed considering sporting discipline, specific issues related to REDs, details of the administered product in terms of quantity and composition, and the outcomes of the experiments. In addition, background information for the review, such as the roles of nutrients, dietary sources, definitions, and general recommendations, was acquired and incorporated using other methods as well, including referencing websites of relevant organisations or citations from related publications. The detailed selection process is illustrated in Figure 1.

## 3. Results

The results were divided into several sections. The first three of them present an introduction describing essential information on LEA, BMD, and menstrual disorders. The next one is a summary of nutritional deficiencies prevalent in various sports among female athletes. The subsequent sections provide an overview on dietary management strategies addressing LEA, BMD, and hormonal disorders.

### 3.1. Low Energy Availability

LEA is one of the three major components of the TRIAD and the main cause of adverse health effects of REDs. Energy availability refers to the amount of energy necessary to sustain essential bodily functions. It is calculated by subtracting exercise energy expenditure (EEE) from the total energy intake throughout the day and subsequently dividing it by fat-free mass (FFM) in terms of kilograms [8,9].

$$Energy\;Availability=\frac{energy\;intake\left[\mathrm{kcal}\right]\;\;-\;\;exercise\;energy\;expenditure\;\left[\mathrm{kcal}\right]}{fat-free\;mass\left[\mathrm{kg}\right]}$$

Athletes’ inadequate knowledge of nutrition is among the various reasons for the incidence of LEA. However, it may also result from the increasing prevalence of eating disorders (EDs), complex medical conditions characterised by incorrect eating habits adversely impacting health and functional ability, e.g., anorexia and bulimia nervosa. Those disorders often affect athletes in weight-class sports or those that emphasise leanness [8,10]. Muia et al. demonstrated that 75% of elite Kenyan runners exhibited ED-related behaviour [11]. Professional dancers with strict dietary restrictions show a lower EA as compared to those following standard diets [12]. A study by Reed et al. reported high levels of body dissatisfaction among female soccer players experiencing LEA [13]. Similar issues were observed in female endurance athletes with amenorrhea as compared to those with normalised menstruation [14].

A universally accepted gold standard for assessing EA has not yet been established. The prevalent methods chosen in research to measure dietary nutrient intake include 24 h interviews (24HR) and food frequency questionnaires. The most commonly applied method, albeit prone to significant underestimation bias, involves the use of food self-reports. Each method comes with its own set of advantages and limitations, and the choice should be tailored to the specific assessment required. The 24HR method is considered one of the more accurate approaches currently available as it has been designed to estimate present nutrient intake while addressing the problem of underestimating energy intake. However, a single dietary test may lack reliability due to intra-personal variations and fluctuations throughout the day. Hence, it is crucial to collect data from at least 3 days of intake, including at least 1 weekday, to provide a more comprehensive dietary assessment [15,16]. Minimising measurement errors is paramount as these errors may stem from various factors, such as differing motivation, memory, and the honesty of study participants. Therefore, it is essential that the individuals tasked with data collection possess the requisite qualifications and skills. They must be adept at meticulously instructing participants on accurately completing dietary diaries. Additionally, they should have the expertise to discern which questions to ask and how to frame them, ensuring thoroughness and reducing the likelihood of omission [17,18,19].

An accurate estimate of the EEE is another component required for calculating the EA. This estimation may vary based on an athlete’s training regimen, performance level, and preferred daily lifestyle choices [20]. The EEE is also susceptible to selection bias and survival bias, which is why it is necessary to conduct studies across various groups of sports [20,21]. Researchers have employed diverse methods to calculate the EEE in their studies. It is important to note that there is not only variability in reporting the EA in different units, but also in the use of what is known as lean body mass (FFM plus essential fat) instead of the FFM (defined as the total body mass minus fat) [20,22]. Some studies have utilised the adjusted EEE (i.e., subtracting the energy cost of sedentary exercise behaviour during the exercise period from the EEE) [8]. Attempts have also been made to use the resting metabolic rate (RMR) and the measured/predicted RMR ratio. Nevertheless, predictive equations have proven excessively variable in both genders, rendering them an unreliable marker at this time [20,22].

Recently, validated questionnaires such as the Low Energy Availability in Females Questionnaire (LEAF-Q) have emerged in research, capable of identifying athletes exhibiting symptoms of LEA [23,24]. Given its association with EDs, combining those tools with athlete-specific ED assessments, such as the Female Athlete Screening Tool (FAST) [25] and the Brief EDs in Athletes Questionnaire (BEDA-Q) [26], could prove valuable. Dervish et al. found that 47% of female endurance runners were at risk of LEA, 40% were at risk of disordered eating (a broad term encompassing a disturbed relationship with food, exercise, and the body, including emotional eating [10]), and 9% met criteria for EDs [23]. Similar results were observed in female athletes by Sharps et al., with 53% at risk of LEA, 44% exhibiting disordered eating, and 6% meeting criteria for EDs [27].

Based on the findings of those studies, it is crucial to detect LEA in athletes because its long-term presence represents only the tip of the iceberg concerning further health consequences. Therefore, ongoing efforts are focused on identifying markers that could more directly reveal the occurrence of LEA. Leptin, total and free T3, insulin-like growth factor 1, urinary LH surge, and markers of bone formation and resorption (carboxy-terminal propeptide of type 1 procollagen) are among the most commonly considered markers [28].

### 3.2. Bone Mineral Density

Age is not the only factor contributing to the loss of bone mass; it can also be experienced among young women. Stress fractures (SFs) typically result from external forces. Under normal circumstances, bones undergo constant remodelling and adapt to various loads. However, abnormal and repetitive loading may lead to the formation of SFs. In such instances, the body is unable to adequately and promptly compensate. Consequently, micro-injuries and fractures occur [29,30]. SFs most commonly affect the lower limb (8–95%) whereas they occur far less frequently in the upper limb (fewer than 10% of SFs) [31]. However, athletes with low BMD face a substantially higher risk of SFs as compared to those with normal BMD. Similar risks emerge when athletes experience nutritional issues related to LEA. Barrack et al. indicated that training for more than 12 h per week was the primary factor contributing to bone stress injury (BSI). The presence of low BMD amplifies this risk 3-fold. When combined with a body mass index <21 kg/m^2^ and amenorrhea, the risk increases 4-fold [32]. This correlation likely stems from low oestradiol levels and insufficient energy supply, leading to an imbalance in bone metabolism. Lappe et al. [33] revealed that calcium and vitamin D supplementation reduced the incidence of SF in female military recruits.

Nutrition plays a pivotal role in ensuring healthy skeletal growth and development, maintaining normal density and thus protecting against conditions such as osteoporosis, bone fragility, and fractures as individuals age. Adequate intake of energy as well as macronutrients and micronutrients—particularly vitamin D, calcium, and phosphorus—is tremendously important [34,35].

### 3.3. Menstrual Disorders

Nutritional and behavioural changes are essential components of the initial treatment for a hypoestrogenic state and LEA to further influence the return of menstruation and support BMD [1]. The prevalence of menstrual disorders in active women ranges from 19% to 54% [36]. Female athletes are commonly diagnosed with functional hypothalamic amenorrhoea (FHA), where there is inhibition of the hypothalamic–pituitary–ovarian axis function and a reduction in gonadoliberin dipeptide (GnRH) secretion. High training overload and stress, which may result in decreased body weight (BW) and insufficient energy intake, are among the most common factors of FHA [37]. Research reveals that the resumption of menstrual cycles is dependent on nutritional status, including achieving and maintaining adequate BW and FFM [38,39]. A balanced diet and improved energy intake are beneficial not only for weight restoration but also for enhancing bone mass and the GnRH secretion [38,40]. De Souza et al. demonstrated that increasing daily energy intake (330 ± 65 kcal/day; 18 ± 4%) could facilitate the return of menstruation in female athletes with oligomenorrhoea and amenorrhoea [41].

### 3.4. Consumption of Energy, Macronutrients, and Specific Micronutrients among Female Athletes

#### 3.4.1. Exercise Energy Expenditure (EEE) and Energy Availability (EA)

In the data retrieved (Table 1), EEE ranged from 272 ± 78 to 1300 ± 293 kcal/day. A lowest EEE of 272 ± 78 kcal/day was reported by De Souza et al. [42] for a group of exercising amenorrheic women. In contrast, a highest result of 1300 ± 293 kcal/day was obtained by Schaal et al. [14] for a group of amenorrheic athletes from the United States. The analysis of the EA data (Table 1) showed that EA ranged from 18 ± 6.6 to 42.5 ± 12.1 kcal/kg FFM per day. Schaal et al. [14] demonstrated a value of 18 ± 6.6 kcal/kg FFM per day. In contrast, the highest value (42.5 ± 12.1 kcal/kg FFM per day) was obtained by Melin et al. [43] in a group of female endurance athletes from Denmark and Sweden. To summarise, in various studies, LEA defined by a suggested cut-off point of 30 kcal/kg FFM/day [2,44] was noted among runners [42,45], endurance athletes [14], artistic gymnasts [46], and dancers [47].

#### 3.4.2. Macronutrients

Besides providing adequate energy, the diet should also cover nutrient requirements [7]. Inadequate **protein** intake has been observed in female athletes participating in sports where a lean body composition is important. This deficiency has also been shown in nearly one in three female soccer players (less than 1.2 g/kg BW) [53,54,55]. Regarding total daily protein consumption, its intake ranged from 13% to 21% (Table 2). The lowest values were documented in three studies [14,46,47], whereas the highest intake was reported by Condo et al. [56] among soccer players. Difficulty in meeting the recommended protein intake of 1.2–2 g/kg BW/day [57] was observed only in a group of artistic gymnasts [46].

**Carbohydrate** consumption (CHO) is a concern among female athletes. The required amount of this macronutrient varies depending on the duration, intensity, and frequency of training sessions [58]. Nevertheless, reported daily CHO intake values ranged from 41% among female soccer players [56] to 62% among runners [42] (Table 2). Considering the values of the standards used by the authors (in their absence, a CHO target from 5 to 7 g/kg/day was selected [57]), insufficient consumption was evident among various groups of athletes, including soccer players [56,59], artistic gymnasts, swimmers [46], and runners [45].

The last but not least important macronutrient in an athlete’s diet is **fat.** Fat intake should follow general guidelines for healthy people (20–35% of total energy intake) and should be considered individually based on training levels (at no less than 20% of total energy intake) [57,60]. Reported daily dietary intake ranged from 22% to 40% (Table 2). The lowest intake (22%) was noted by De Souza et al. [42], while the highest (40%) was noted by Jakše et al. [46] in a group of young Slovenian artistic gymnasts. However, no significant issues in fulfilling fat intake requirements were identified in either study.

#### 3.4.3. Selected Micronutrients

A properly balanced diet, encompassing appropriate proportions of macronutrients and a careful selection of food products rich in micronutrients, is vital in preventing nutritional deficiencies and associated health consequences [3,5].

In the reviewed studies (Table 3), daily **vitamin D** intake ranged from 1.69 µg (among Polish female soccer players [59]) to 8.3 µg (in runners from the USA [48]). Therefore, none of the groups of athletes in the analysed studies demonstrated coverage of the standard for this vitamin. **Calcium** (**Ca**)**,** closely related in its functions to the vitamin D, exhibited a range of intake from 608 mg/day (in athletes with disabilities from Korea [61]) to 1532 mg/day (among long-distance Lithuanian runners [62]) (Table 3). **Phosphorus** (**P**) intake plays a significant role in the adequate absorption of Ca. What is also important is the ratio of both minerals [63,64]. P intake varied between 702 mg/day [61] and 2103 mg/day [62] (Table 3). The **Ca/P ratio** calculated on the basis of the provided data ranged from 0.555 [59] to 0.867 [61] (Table 3). Most of the groups of examined athletes experienced a problem with Ca coverage from the diet. The exceptions were athletes of certain disciplines in the study by Baranauskas et al. [62], McCormack et al. [48], and Soric et al. [65]. The situation was similar for maintenance of a proper Ca/P ratio; only long-distance runners were close to balancing both components [62].

**Magnesium** (**Mg**) and **zinc** (**Zn**) are crucial in sports nutrition. Studies have indicated that Mg intake ranged between 245 mg/day [59] and 595 mg/day [62] (Table 3). In the case of Zn, consumption varied between 6.0 mg/day (in Brazilian swimmers with eating disorders [66] as well as disabled athletes [61]) and 19 mg/day [62] (Table 3). **Iron** (**Fe**) is also vital for sustaining high-level aerobic capacity [67,68]. Its intake in the reviewed research ranged from 8 mg/day [61] to 27 mg/day [62] (Table 3). In the case of Mg, non-coverage of the average requirement was noted for soccer players [59], artistic gymnasts, swimmers [46], ballerinas, rhythmic gymnasts [65], and runners [45]. There was a similar issue about Zn in the group of swimmers [66] and disabled female athletes [61], and the same was true of Fe among disabled athletes [61] as well as swimmers and artistic gymnasts [46].

**Table 2:** Macronutrient intake among female athletes.

Macro- Nutrients	Norms		Results			Sports Discipline
	% of Energy or g/Day	g/kg BW	% of Energy	g/Day	g/kg BW	
Proteins	50 g/day	1.2–2.0	17 *	56	n/d	disabled athletes [61]
	n/d	1.2	14 * ^ArtGym^ 13 * ^Swim^	55 * ^ArtGym^ 73 * ^Swim^	1.0 ± 0.2 ^ArtGym^ 1.2 ± 0.2 ^Swim^	artistic gymnastics, swimming [46]
	n/d	n/d	16 * ^ExAnov^ 14 * ^ExOvul^ 14 * ^ExLPD^	58 * ^ExAnov^ 68 * ^ExOvul^ 70 * ^ExLPD^	n/d	running [42]
	n/d	n/d	17	n/d	n/d	ballet [69]
	n/d	1.2–2.0	15 *	71	1.4 ± 0.6	running [45]
	83 g/day	1.4–1.7	19 *	72	1.2 ± 0.44	soccer [59]
	n/d	1.2–1.7	13 *	81	1.3 ± 0.3	dancing [47]
	n/d	n/d	13 ^AM^ 15 ^EU^	68 * ^AM^ 83 * ^EU^	n/d	endurance sports [14]
	n/d	1.2–2	21	98	1.5 ± 0.5	soccer [56]
	71 g/day	1.2–1.7	17	116 ± 26	2.0 ± 0.5	endurance sports [43]
	n/d	1.3–1.7	n/d	80 ^Interm^ 77 ^Advanced^ 82 ^Elite^	1.3 (1.2–1.5) ^Interm^ 1.4 (1.2–1.6) ^Advanced^ 1.4 (1.2–1.9) ^Elite^	climbing [50]
Carbohydrates	n/d	8–12	47 * ^ArtGym^ 54 * ^Swim^	181 * ^ArtGym^ 305 * ^Swim^	3.3 ± 0.8 ^ArtGym^ 5.1 ± 1.6 ^Swim^	artistic gymnastics, swimming [46]
	n/d	5–7 ^Up to 1 h exercise/day^ 6–10 ^1–3 h exercise/day^	41 *	192	3.0 ± 0.8	soccer [56]
	296 g/day	5–7	53 *	199	3.3 ± 1.2	soccer [59]
	130 g/day	n/d	49 *	164	n/d	disabled athletes [61]
	n/d	n/d	56	218*	n/d	ballet [69]
	n/d	n/d	62 * ^ExAnov^ 60 * ^ExOvul^ 57 * ^ExLPD^	229 * ^ExAnov^ 285 * ^ExOvul^ 288 * ^ExLPD^	n/d	running [42]
	n/d	6–10	53 *	255	4.9 ± 2.1	running [45]
	n/d	n/d	52 ^EU^ 57 ^AM^	286 * ^EU^ 299 * ^AM^	n/d	endurance sports [14]
	n/d	5–7	52 *	313	5.0 ± 1.0	dancing [47]
	353 g/day	6–10	53	369	6.4 ± 1.6	endurance sports [43]
	n/d	3–7	n/d	282 ^Interm^ 228 ^Advanced^ 253 ^Elite^	4.6 (4.0–5.4) ^Interm^ 4.2 (3.4–5.3) ^Advanced^ 4.2 (4.2–5.8) ^Elite^	climbing [50]
Fats	15–30%	n/d	34 *	52 *	n/d	disabled athletes [61]
	n/d	n/d	26	45 *	n/d	ballet [69]
	49.2 g/day	n/d	28	47	0.78 ± 0.39	soccer [59]
	n/d	n/d	22 * ^ExAnov^ 26 * ^ExOvul^ 29 * ^ExLPD^	36 * ^ExAnov^ 54 * ^ExOvul^ 64 * ^ExLPD^	n/d	running [42]
	30–40%	n/d	40 ^ArtGym^ 38 ^Swim^	n/d	n/d	artistic gymnastics, swimming [46]
	n/d	n/d	28 ^EU,AM^	68 ^EU^ 65 ^AM^	n/d	endurance sports [14]
	n/d	n/d	32 *	69	n/d	running [45]
	20–35%	n/d	35	72	n/d	soccer [56]
	<30%	n/d	34	92	1.5 ± 0.4	dancing [47]
	20–35%	n/d	30	93	1.6 ± 0.5	endurance sports [43]
	n/d	n/d	n/d	70 ^Interm^ 60 ^Advanced^ 68 ^Elite^	1.2 (1.0–1.6) ^Interm^ 1.1 (0.9–1.3) ^Advanced^ 1.2 (0.8–1.2) ^Elite^	climbing [50]

Values are expressed as mean or median. For the data presented in terms of g/kg, the resulting figures are expressed in terms of the mean ± standard deviation or median (interquartile range). * In cases where the author presented a parameter in a different unit than that established in this review, general recalculations were performed where feasible. Abbreviations: AM—amenorrheic, ArtGym—artistic gymnasts, BW—body weight, EU—eumenorrheic, ExOvul—exercise/ovulatory, ExLPD—exercise/luteal phase deficiency, ExAnov—exercise/anovulatory, Interm—Intermediate, n/d—no data, Swim—swimmers.

**Table 3:** Micronutrient intake among female athletes.

Micronutrient	Norm	Results			Sports Discipline
Vit. D (µg)	10 [61] 15 [45,60] 20 [46] $\bar{x}$ = 15	1.69			soccer [59]
		2.5 (1.3–5.9) ^Interm^	2.66 (0.9–4.1) ^Elite^	3.9 (1.3–7.2) ^Advanced^	climbing [50]
		2.6			disabled athletes [61]
		2.8 ± 2.2 ^HCycl^	3.2 ± 3.1 ^Swim^	3.4 ± 3.1 ^Rowers^	endurance sports [62]
		3.4 ± 2.7 ^LDRun^	3.7 ± 2.5 ^Skiers^	3.7 ± 3.0 ^BAthl^	
		4.5 ± 0.4			running [45]
		5.5 ± 9.6 ^Gym^	3.5 ± 3.5 ^Swim^		artistic gymnastics, swimming [46]
		8.3 ± 7.2			running [48]
Ca (mg)	700 [61] 800 ^19–50 y^/1000 ^>50 y^ /1100 ^10–18 y^ [59,60] 1200 [46] 1300 [45] $\bar{x}$ = 1000	608			disabled athletes [61]
		629 ± 274 ^Gym^	806 ± 228 ^Swim^		artistic gymnastics, swimming [46]
		646 ± 290			soccer [59]
		703 (605–817) ^Advanced^	706 (537–1097) ^Interm^	857 (753–1107) ^Elite^	climbing [50]
		706 (332–1542) ^DE+ (15–19 y)^	819 (93–1738) ^DE+ (11–14 y)^		swimming [66]
		843 (269–2305) ^DE− (11–14 y)^	909 (329–2563) ^DE– (15–19 y)^		
		925 ± 545			soccer [56]
		1000 ± 504 ^Swim^	1066 ± 407 ^BAthl^	1117 ± 543 ^Skiers^	endurance sports [62]
		1163 ± 484 ^HCycl^	1398 ± 399 ^Rowers^	1532 ± 1342 ^LDRun^	
		1013 ± 448 ^ArtGym^	1052 ± 577 ^RhytGym^	1680 ± 304 ^Ballet^	gymnastics, ballet [65]
		1046 ± 58.9			running [45]
		1395 ± 684			running [48]
P (mg)	580 ^10–18 y^/1050 ^>18 y^ [59,60] 700 [61] 1250 [45,46] $\bar{x}$ = 900	702			disabled athletes [61]
		924 ± 192 ^ArtGym^	1236 ± 188 ^Swim^		artistic gymnastics, swimming [46]
		1165 ± 357			soccer [59]
		1203 (1044–1451) ^Advanced^	1370 (1192–1546) ^Interm^	1535 (1400–1739) ^Elite^	climbing [50]
		1256 ± 563 ^RhytGym^	1290 ± 567 ^ArtGym^	1353 ± 312 ^Ballet^	gymnastics, ballet [65]
		1341 ± 72			running [45]
		1569 ± 549			soccer [56]
		1646 ± 321 ^LDRun^	1740 ± 732 ^Skiers^	1816 ± 552 ^HCycl^	endurance sports [62]
		1841 ± 520 ^BAthl^	1865 ± 650 ^Swim^	2103 ± 546 ^Rowers^	
Ca/P	0.96 [46] 1 [61] 1.04 [45] 1.05/1.37 [59] $\bar{x}$ = 1	0.555			soccer [59]
		0.589			soccer [56]
		0.536 ^Swim^	0.579 ^BAthl^	0.641 ^HCycl^	endurance sports [62]
		0.642 ^Skiers^	0.665 ^Rowers^	0.931 ^LDRun^	
		0.680 ^ArtGym^	0.652 ^Swim^		artistic gymnastics, swimming [46]
		0.780			running [45]
		0.785 ^ArtGym^	0.838 ^RhytGym^	0.863 ^Ballet^	gymnastics, ballet [65]
		0.867			disabled athletes [61]
Mg (mg)	280 [61] 255/300 [59] 350 [46] 360 [45] $\bar{x}$ = 310	245			soccer [59]
		292 ± 80 ^ArtGym^	333 ± 79 ^Swim^		artistic gymnastics, swimming [46]
		301 ± 115 ^RhytGym^	309 ± 64 ^Ballet^	347 ± 183 ^ArtGym^	gymnastics, ballet [65]
		317 (245–357) ^Advanced^	383 (322–505) ^Interm^	473 (411–510) ^Elite^	climbing [50]
		351 ± 18			running [45]
		368 ± 138			soccer [56]
		448 ± 191 ^HCycl^	464 ± 150 ^BAthl^	480 ± 226 ^Skiers^	endurance sports [62]
		493 ± 163 ^Rowers^	503 ± 221 ^Swim^	595 ± 335 ^LDRun^	
Zn (mg)	7 [46,59] 8 [61] 9 [45] $\bar{x}$ = 8	6 (1–14) ^DE+ (15–19 y)^	7 (1–16) ^DE+ (11–14 y)^		swimming [66]
		7 (2–17) ^DE− (11–14 y)^	10 (2–120) ^DE− (15–19 y)^		
		6			disabled athletes [61]
		7 ± 2 ^ArtGym^	9 ± 6 ^Swim^		artistic gymnastics, swimming [46]
		8 ± 3			soccer [59]
		10 (7–12) ^Advanced^	11 (10–12) ^Interm^	12 (12–13) ^Elite^	climbing [50]
		10 ± 2 ^Ballet^	11 ± 9 ^ArtGym^	12 ± 7 ^RhytGym^	gymnastics, ballet [65]
		12 ± 1			running [45]
		12 ± 4			soccer [56]
		13 ± 2 ^LDRun^	14 ± 5 ^Skiers^	15 ± 4 ^BAthl^	endurance sports [62]
		15 ± 5 ^HCycl^	16 ± 6 ^Swim^	19 ± 6 ^Rowers^	
Fe (mg)	8 [59] 8/14 [61] 15 [45,46] $\bar{x}$ = 11	8			disabled athletes [61]
		9			soccer [59]
		9 ± 4 ^ArtGym^	14 ± 7 ^Swim^		artistic gymnastics, swimming [46]
		11 ± 3 ^Ballet^	15 ± 7 ^ArtGym^	15 ± 9 ^RhytGym^	gymnastics, ballet [65]
		12 ± 3			soccer [56]
		12 (11–16) ^Advanced^	17 (14–17) ^Interm^	20 (18–21) ^Elite^	climbing [50]
		13 (4–23) ^DE+, DE− (11–14 y)^	13 (6–27) ^DE+ (15–19 y)^	16 (7–35) ^DE− (15–19 y)^	swimming [66]
		16 ± 1			running [45]
		17 ± 5			middle- and long-distance running, race walking [51]
		20 ± 4 ^LDRun^	24 ± 7 ^BAthl^	26 ± 11 ^Skiers^	endurance sports [62]
		27 ± 10 ^Swim^	27 ± 12 ^HCycl^	27 ± 7 ^Rowers^	

Values are expressed as mean (if given: ± standard deviation) or median (interquartile range). To compare the authors’ reported results with established norms, the values presented in their respective studies were taken into account. In instances where the standard employed by the authors was unspecified, the results were compared with averaged values. If the author did not specify the Ca/P ratio, it was calculated on the basis of the extracted data. Abbreviations: ArtGym—artistic gymnasts, BAthL—biathletes, DE+—disordered eating, DE−—without disordered eating, HCycl—highway cyclists, Interm—Intermediate, LDRun—long-distance runners, RhytGym—rhythmic gymnasts, Swim—swimmers, y—years.

### 3.5. Nutritional Management of TRIAD/REDs

#### 3.5.1. Overall Approach

The key approach to TRIAD/REDs nutritional treatment should be holistic; however, it is important to be certain to follow the right steps. Primarily, it is necessary to take care of the appropriate energy density. After providing caloric requirements, the next integral element should be the proper balancing of macronutrients and composing the diet to match the athlete’s need for the necessary micronutrients (vitamins and minerals). Next, the timing of intake throughout the day (before, during and after exercise) and type, length, and intensity of exercise should be optimised. For optimal nutritional care of a female athlete, it is necessary to consider not only her training schedule but also the hormonal fluctuations experienced during respective phases of the menstrual cycle [3].

#### 3.5.2. Energy Requirement

LEA is defined to occur when the result is below 30 kcal/kg FFM per day [44]. In the long term, such an insufficient amount may lead to adverse health effects (including interference with reproductive function and bone metabolism) as well as impairing athletic performance [44,70]. The optimal and physiological cut-off point is 45 kcal/kg FFM per day. Intermediate values (30–45 kcal/kg FFM/day) are amounts that can be tolerated for a limited period of time in female competitive athletes who would like to reduce BW with a properly designed and balanced diet and training [2,9,71]. Nevertheless, it is important to remember that those are not specific diagnostic values and there has been no established definitive clinical threshold for EA. The values may vary based on individual variations [2]. The key is to design meal plans that not only enhance the nutrient and energy density of meals without substantially increasing their volume but also take into consideration the athlete’s dietary preferences, lifestyle, training regimen, and competition schedules. Cooperation with a psychologist may significantly boost motivation to implement nutritional changes and achieve a balanced diet. Most importantly, the diet should include high-energy-density food, such as dried fruits; dairy drinks fortified with proteins, calcium, and vitamin D; and products rich in essential fatty acids, such as avocado, fish, vegetable oils, nuts, tahini, and chia seeds. Moreover, since athletes commonly experience gastrointestinal issues, it is advisable to recommend an increase in the frequency of small-volume meals [1,3,57,72,73].

#### 3.5.3. Macronutrient Requirements

The recommended daily **protein** intake for female athletes, irrespective of the menstrual phase, should fall within the range outlined in current sports guidelines (1.2–2.0 g/kg BW/day [57] or even 1.8–2.2 g/kg BW/day [74]). In order to maintain or build up FFM in the presence of LEA, a higher intake of protein is recommended (approximately 2 g/kg BW/day), due to its crucial role in muscle protein synthesis and tissue repair [75,76]. However, researchers also suggest that protein requirements in women exercising 1.5 h/day should be at least 1.6 g/day during their follicular phase [77]. During the luteal phase, there is notably higher catabolism of this macronutrient as compared to the follicular phase. To sustain optimal muscle protein synthesis and strength, it is advisable to consume 10 g of essential amino acids (equivalent to 15–25 g of high-biological-value protein) within 2 h after training [74]. If the aim is to prioritise muscle mass growth and repair over using protein oxidation for fuel, it is crucial to ensure an appropriate balance between CHO and energy intake in relation to energy expenditure to accurately address the athlete’s requirements [78].

An adequate amount of **CHO** is essential to fuel the brain, support both aerobic and anaerobic metabolism, and maintain hormonal balance [79]. Moreover, it significantly influences performance and aids in recovery [58]. CHO requirements depend on the duration, intensity and frequency of training sessions as well as weather conditions. If an athlete conducts low-intensity training, 3–5 g/kg BW/day is enough. However, for moderate physical activity (more than 1 h/day), the requirement increases to at least 5 to 7 g/kg BW/day. For endurance training (1–3 h/day), 6–10 g/kg BW/day is recommended, while for even more intense exercise (>4–5 h/day), up to 8 to 12 g/kg BW/day [57]. Nevertheless, those are general guidelines for both female and male athletes. However, women in the follicular phase of the menstrual cycle benefit from increased glycogen stores (via a CHO load of 8.4–9.0 g/kg BW) as compared to the luteal phase, where glycogen storage is higher and CHO oxidation is lower [58,74,80]. Initially CHO at a rate of 30–60 g/h during training may help counterbalance the menstrual cycle’s effect on glucose kinetics and exercise metabolism. This approach can also aid in minimising the likelihood of gastrointestinal disorders. Furthermore, rapid intake of CHO (at a rate of 1.2 g/kg BW) is important after prolonged physical workouts [74].

**Fats** play a crucial role in metabolic and hormonal sustainability, in addition to replenishing intra-muscular triglyceride reserves and maintaining energy balance, thereby holding tremendous importance for female athletes [79,81]. In a study by Hausswirth et al. [53] women expended more fat during exercise as compared to men, due to a lower respiratory exchange ratio (RER). In addition, women exhibited enhanced lipolytic activity during prolonged moderate-intensity physical activity. Oestrogen enhances lipid peroxidation during athletic endeavours, resulting in elevated levels of free fatty acids. Manipulating the quantity and source of dietary fat may impact the levels of several anabolic hormones in blood, consequently affecting both body composition and efficiency [81]. Athletes should avoid fat intake below 20% of energy. This practice may reduce dietary diversity and cause deficiencies in fat-soluble vitamins and essential fatty acids, particularly *n-*3 [57].

#### 3.5.4. Micronutrient Requirements

All nutrients are essential and have a key impact on an athlete’s health. However, it is crucial to cover the ones (vitamin D, calcium, phosphorus, magnesium and zinc) that influence BMD as well. Proper selection of food groups and inclusion of fortified products may prevent nutritional deficiencies [34,35]. Meals should be composed to include products that support absorption and minimise interactions with products that reduce bioavailability (Table 4). On the other hand, when it is not possible to ensure the intake of adequate amounts of micronutrients from the diet, e.g., due to excessive needs, after consultation with a specialist, appropriate supplementation with preparations containing the most bioavailable forms should be implemented (Table 4).

The benefits of **vitamin D** supplementation are widely recognised, particularly for bone health and immune system support. For athletes, its significance extends to aiding recovery from injuries, optimising performance, and maintaining normal neuromuscular function [57,79]. In cases of deficiency, the absorption of calcium and phosphorus may decrease by up to 15% and 60%, respectively. Notably, a study involving Navy recruits observed a reduction in SF with the supplementation of 800 IU of vitamin D and 2000 mg of calcium in [33]. Additionally, more than three-quarters of injuries among swimmers and divers were associated with decreased levels of 25-hydroxy vitamin D, implying that a preventive dose of 4000 IU might be beneficial [82]. Foods particularly abundant in vitamin D include eggs, dairy products, such as milk and cheese, and fatty fish, such as salmon, herring, and mackerel (Table 4) [60,83,84,85]. To increase the absorption of this vitamin from the gastrointestinal tract, meals should contain fats and vitamin E, while polyunsaturated and long-chain fatty acids and phytosterols may have a diminishing effect [86,87,88,89]. Cholecalciferol represents the most readily absorbed form of vitamin D. However, it is crucial to note that endogenous synthesis, which occurs through exposure to sunlight, remains extremely important [89,90,91,92].

Maintaining optimal bone density also requires adequate **Ca** intake. A study found that 85% of female runners with elevated bone turnover did not meet the recommended intake for Ca [93]. Currently, the recommendation stands at 1500 mg/day for athletes with amenorrhea, nutritional disorders, or early risk of osteoporosis [94]. Adequate calcium levels can lower the likelihood of skeletal system injuries. A dose of 2000 mg reduced the number of fractures [33]. Delayed menarche may increase the risk of low BMD due to the effect of oestrogen on Ca transfer to bone [95]. It is essential to incorporate calcium into the diet as research suggests that the beneficial effects on BMD become insignificant once supplementation is discontinued [96]. The main dietary sources of this element include milk and dairy products, as well as plant products such as parsley, kale, spinach, and beans (Table 4) [60,83,97,98]. Nutrients that notably enhance the absorption of Ca are lactose and vitamin D_3_. Conversely, it is important to be mindful of foods high in fibre and phosphorus, oxalic acid, and phytic acid, as they can hinder calcium absorption [83,99,100,101]. When considering supplementation, suitable forms are calcium carbonate, citrate, and gluconate [97,101,102]. For athletes at risk for low calcium levels, a daily intake of 1500 mg is recommended to maintain optimal bone health, particularly relevant for women with LEA and menstrual disorders [103]. It is worth remembering that the intestine cannot absorb more than 500 mg of calcium at one time, necessitating the distribution of the element throughout the day [104].

**P** holds particular significance in the synthesis of adenosine 5′-triphosphate (ATP) and other high-energy compounds, such as adenosine diphosphate, guanosine triphosphate, and phosphocreatine. Thus, it plays a key role as far as the functions of skeletal muscles are concerned, ensuring their normal contractility. Furthermore, phosphorus is important for neuromuscular conduction. Additionally, it contributes to maintaining a normal acid–base balance [105]. It has been proven that a high dietary supply of P may contribute to endocrine disruption related to parathormone (PTH), leading to lower blood Ca levels. And conversely, a high dietary Ca supply is known to hinder phosphate absorption, consequently lowering blood PTH levels [63,64]. Elevated blood P concentrations were frequently observed in the athletes studied; these can potentially be attributed to high dietary phosphorus intake and rhabdomyolysis associated with intense exercise [106]. Phosphorus is most abundant in legumes, eggs, fish, offal, and wholegrain bread (Table 4). Optimal absorption of this element occurs with organic phosphate esters and ionised inorganic forms [107,108,109,110,111]. Factors that may restrict absorption include vitamin D deficiency and a high dietary Ca intake [107,109].

**Mg** is equally relevant for athletes to ensure proper oxygen uptake and electrolyte balance, as well as having an impact on the endocrine system [94,112]. Research indicates that athletes may require up to 20% higher magnesium intake as compared to standard population guidelines [112]. Studies have revealed that a significant proportion of women from a variety of sports have reported inadequate magnesium intake, with silhouette sports being among the most vulnerable due to restricted energy intake [78,112,113]. Deficiency in magnesium may elevate the oxidative costs associated with training, a factor relevant to endurance performance [78]. To maximise dietary Mg intake, foods such as cereals, legumes, nuts, cocoa, rennet cheeses, potatoes, and bananas are beneficial (Table 4) [60,83]. For optimal absorption, it is advised to avoid products containing phytic acid, Ca, and P [114,115,116]. Instead, magnesium should be consumed alongside food products rich in vitamin B_6_ and substances that lower the pH of the digestive tract [115,116,117]. Magnesium citrate, aspartate, and lactate are recommended forms of Mg supplementation [114,118].

**Zn** is significant for the proper conduct of metabolic processes and muscle function. Although its deficiency is rare, in athletes, it is most commonly lost through sweat and skeletal muscle breakdown [68,119,120]. In addition, the combination of LEA and a vegan diet may exacerbate this condition, due to this diet’s low content and low bioavailability of food products of plant origin. Iron-rich food products also serve as important sources of Zn [68,121]. It is important to note that during zinc supplementation, absorption of Fe and copper may be impaired. Eskici et al. [122] observed that supplementation of Zn at a dose of 220 mg/day for 4 weeks did not lead to increased urinary excretion of Mg, Ca, P, or copper. However, despite this observation, it is still advisable to measure those factors in the case of zinc administration. Dietary sources rich in Zn include meat products, especially liver, as well as eggs, buckwheat groats, and wholegrain bread [60,123]. The absorption of Zn may be impaired by the presence of oxalic and phytic acids, fibre, ethanol, and other minerals, such as Fe or cadmium (Table 4). Citric acid and a diet abundant in animal protein may facilitate Zn absorption in the intestines [99,121,123,124,125]. Gluconate, citrate, and picolinate constitute the most commonly utilised chemical forms for Zn supplementation [99,126].

Due to significantly increased loss of **Fe** among athletes (30–70%), deficiency is a common diagnosis. This deficiency may adversely affect athletes’ capabilities, e.g., by reducing performance time, lowering VO_2_ max (maximal oxygen consumption), diminishing energy efficiency, and impeding the ability to work out at an optimal level [127,128]. However, studies show that Fe supplementation may have a beneficial effect on iron status as well as athletic performance, particularly in individuals with reduced iron levels [129,130]. Meat products, particularly liver and kidney, are recognised for their high Fe content (Table 4) [60]. The haem Fe available in the diet is the most easily absorbed by the human body. For female athletes, it is suggested that Fe intake should be increased by up to 70% of the estimated average requirement [131]. Meanwhile, if a woman belongs to a risk group (e.g., vegetarians and distance runners), consumption higher than the recommended dietary allowance (>18 mg/day) should be considered [57,132]. Other well-absorbed forms of Fe include ferrous sulphate, gluconate, and citrate [133,134,135]. Intestinal absorption of this element may be readily impaired by phytic and oxalic acids, insoluble fibre fractions, polyphenols, and large intakes of Zn [136,137,138]. To facilitate Fe absorption, it is worth ensuring the presence of ascorbic or lactic acid in the diet [133,136,139,140,141].

**Table 4:** Dietary sources and bioavailability of micronutrients.

Micronutrient	Dietary Sources	Bioavailability Enhancement	Bioavailability Impairment	Best Bioavailable Forms
Vit. D [60,83,84,85,86,87,88,89,90,92]	fat-rich fish (salmon, herring, mackerel), eggs, milk, dairy products and cheese	fat-rich meals, vitamin E	polyunsaturated fatty acids, phytosterols, long-chain fatty acids	“exposure to sunlight”, cholecalciferol
Ca [60,83,97,98,99,100,101,102]	milk, milk-based foods, kale, parsley leaves, spinach, bean seeds	lactose, vitamin D, phosphopeptides	oxalic acid (spinach, rhubarb, beans), phytic acid (seeds, nuts, grains, certain raw beans and soy isolates), insoluble fibre fractions, high phosphorus content	calcium carbonate, gluconate
P [83,107,108,109,110,111]	rennet cheese, buckwheat groats, fish, offal, meat, wholegrain bread, legumes, eggs	phosphorus from animal products, activation of phytases in plant products (sprouting process, soaking legumes, using sourdough to bake bread)	calcium, vitamin D deficiency	organic phosphate esters, ionised inorganic forms
Mg [60,68,83,114,115,116,117,118]	cereals, legumes, nuts, cocoa, dark chocolate, rennet cheese, potatoes, bananas, drinking water	fermentation of soluble fibre fractions, acidic pH, vitamin B6- pyridoxine	phytic acid, phosphates, calcium	magnesium carbonate, citrate, aspartate, hydroaspartate, lactate
Zn [60,99,121,123,124,125,126]	meat, liver, rennet cheese, dark bread, buckwheat groats, eggs	citric acid, animal protein	phytic acid, oxalic acid, insoluble fibre fractions, alcohol, iron, cadmium	zinc gluconate, citrate, picolinate
Fe [60,68,128,133,134,135,136,137,138,139,140,141]	meat, liver, kidney, parsley, legumes, eggs	ascorbic acid, lactic acid, fermented products	phytic acid, oxalic acid, insoluble fibre fractions, plant protein, polyphenols	haem iron, ferrous sulphate, gluconate, ferric citrate, sulphate

## 4. Conclusions

Disorders that occur among female athletes can be severe and affect an ever-growing number of women. The problem of inadequate EA and unmet requirements for protein and carbohydrates within the diet has been observed in various sporting disciplines among women. Furthermore, inadequate vitamin D intake was noted in all the groups of athletes studied. Deficiency was also reported for in average intake of Ca, Mg, Ca/P ratio, Zn and Fe. Low energy availability, low bone density, and menstrual dysfunctions require appropriate therapeutic management, with dietary strategies playing a pivotal role. Nutrition is also crucial as a preventive measure. Therefore, cooperation within an interdisciplinary team consisting not only of a physician but also a nutritionist, physiotherapist, and psychologist is imperative. Further scientific studies assessing this comprehensive approach are also required. This is vital for adapting nutritional interventions aimed at enhancing the physical performance of female athletes and effectively preventing TRIAD/REDs. However, it should not be forgotten that health complications caused by LEA may also affect men.

## Figures and Tables

**Table 1:** Exercise energy expenditure (EEE) and energy availability (EA) values among female athletes.

Parameter	Results	Sports Discipline
EEE (kcal/day)	272 ± 78 ^ExAnov^ 480 ± 53 ^ExOvul^ 494 ± 64 ^ExLPD^	running [42]
	591 ± 95	running [11]
	600 ± 237	running [45]
	800 ± 132 ^EU^ 1300 ± 293 ^AM^	endurance sports [14]
	921 ± 256	running [48]
	940 ± 450	endurance sports [43]
EA (kcal/kg FFM per day)	18.8 ± 3.2 ^ExAnov^ 23.3 ± 1.6 ^ExOvul^ 26.5 ± 1.8 ^ExLPD^	running [42]
	18 ± 6.6 ^AM^ 29 ± 4.8 ^EU^	endurance sports [14]
	23 ± 3 ^ArtGym^ 33 ± 10 ^Swim^	artistic gymnastics, swimming [46]
	26 ± 13	dancing [47]
	29.6 ± 17.4	running [45]
	30.7	running [49]
	31.6 (21.2–37.6)	climbing [50]
	33 ± 7	middle- and long-distance running, race walking [51]
	36.5 ± 4.5	running [11]
	37 ± 21	running [48]
	39.6 (35.3–43.9)	endurance sports [52]
	42.5 ± 12.1	endurance sports [43]

Values are expressed as mean (if given: ± standard deviation) or median (interquartile range). Abbreviations: AM—amenorrheic, ArtGym—artistic gymnasts, EA—energy availability, EEE—exercise energy expenditure, EU—eumenorrheic, ExOvul—exercise/ovulatory, ExLPD—exercise/luteal phase deficiency, ExAnov—exercise/anovulatory, FFM—fat-free mass, Swim—swimmers.

## Data Availability

Not applicable.
